# Coconut tree modeling based on abiotic factors and modified cosserat rod theory

**DOI:** 10.1186/s13007-025-01379-4

**Published:** 2025-05-18

**Authors:** Sakthiprasad Kuttankulangara Manoharan, Rajesh Kannan Megalingam

**Affiliations:** Department of Electronics and Communication Engineering, Amrita Vishwa Vidyapeetham, Amritapuri, India

**Keywords:** Abiotic factors, Biomechanics, Coconut tree climber, Cosserat rod theory, Sensor metadata, Tree measurement

## Abstract

The biomechanics of growing trees, particularly coconut trees, are intricate due to various abiotic factors such as sunlight, wind, gravitropism, and cultivation practices. Existing structural growth models fail to capture the unique characteristics of coconut trees, which lack branches and have large crown leaves. This research introduces a novel coconut tree modeling approach, integrating abiotic factors and modified Cosserat rod theory. Factors like sunlight availability, wind speed, cultivation practices, and gravitropism influence coconut tree growth rates. The model encompasses both primary and secondary growth processes. Primary growth is influenced by gravitropism, sunlight availability, and wind effects, while secondary growth is determined by variations in trunk diameter. Additionally, the model incorporates the diameter at breast height to accommodate cultivation practice variations. Comparisons between the proposed model, classical rod theory, and biomechanics growth models reveal that the proposed model aligns more closely with real-time data on spatial and temporal growth characteristics. This research marks the first attempt to model coconut tree growth considering abiotic factors comprehensively. In summary, this study presents a pioneering coconut tree growth model that integrates abiotic factors and modified Cosserat rod theory. By considering unique features of coconut trees and environmental influences, the model offers more accurate predictions compared to existing approaches, enhancing our understanding of coconut tree biomechanics and growth patterns. Coconut tree modeling has diverse applications in precision agriculture, automated harvesting, tree health monitoring, climate change analysis, urban planning, and the biomass industry, helping optimize yield, resource management, and sustainability. It also plays a crucial role in genetic research, disaster preparedness, and risk assessment, enabling advancements in robotics, environmental conservation, and industrial applications for improved productivity and resilience.

## Introduction

Coconut is economically significant in 86 countries [1]. The unique growth of coconut trees, influenced by abiotic factors such as sunlight, wind, and soil properties, affects their size, shape, trunk diameter, and growth direction [2, 3]. Tree modeling methods include procedure-based, drawing-based, and data-based approaches. Procedure-based methods rely on existing growth rules but require proficiency and manual effort [4]. Drawing-based models depend on user knowledge and are common in gaming and urban modeling. Data-based modeling, an empirical approach, uses measured data from sensors like cameras, accelerometers, and LiDARs [5]. Tree morphology, agronomy, crop science, and physiology studies provide insights into tree health and growth factors [6]. Modeling trees is complex due to variable morphology and abiotic influences. Growth is categorized into primary (height/length) and secondary (trunk diameter). The shape advancement of maturing trees is related to biomechanical responses [7]. Tropism significantly affects growth rate, shape, and direction [8]. Biomechanical processes continuously influence tree maturation and stress variations [9]. Tree measurement methods are either destructive, involving tree removal for detailed data, or non-destructive, using sensors like LiDAR for spatial assessments [10]. Image processing and laser scanning are viable for measuring coconut tree parameters, though climatic dependencies limit accuracy [11].

Early plant biomechanics research relied on the pipe model theory, treating tree trunks as a network of cylindrical conduits [12]. Shinozaki et al. validated the pipe model [13], but it oversimplifies tree physiology, limiting its suitability for coconut trees. Classical rod theory, used by Valentine [14] and Rennolls [15], improves spatial resolution and physiological representation but lacks shear deformation considerations. Berninger et al. [16] developed a mathematical model for leaf distribution in Scot’s pine. Savage et al. [17] introduced plant network scaling to predict sap flow. Cosserat rod theory, incorporating rotational and shearing deformations, offers better tree modeling potential [18]. Biomechanical-based models integrate growth rules, mechanical properties, and environmental effects. Structural analysis [19], wind load assessments [20], and failure predictions [21] are key aspects in tree trunk modeling. Evaluation of tree biomechanical properties was explored in [22, 23]. Some studies model root systems by analyzing soil-root interaction [24], load-bearing capacity [25], and growth morphology [26]. Response to environmental factors in root growth has also been studied [27], Space and light variation in Fagus sylvatica L trees using biomechanics [28] and Effect of dimensional variation on behaviour of trees based on the biomechanics [29]. However, biomechanical models require extensive input data and species-specific calibration, limiting generalizability. Coconut trees, with their straight trunks and large leaves, do not conform to traditional biomechanical models.

Tree measurement methods include destructive and non-destructive techniques. Traditional approaches use calipers and measuring tapes, while modern methods employ LiDAR, aerial imagery, and digital photography. Sun et al. [31] proposed a QR code-based DBH measurement method with two-angle sensors. Laser scanning enables 3D reconstruction for tree modeling [32, 33], but single-sensor setups fail to capture spatial growth characteristics accurately. A multi-beam flash LiDAR sensor for tree vibration analysis was proposed in [34]. Wind dynamics and tree motion in maritime pine forests were studied in [35]. Euler–Bernoulli tapered column models identified tree vibration modes [36], effective for coniferous but less so for broadleaf trees. Infrared motion tracking was applied to pole-like trees [37], while kinetic and potential energy analysis improved tree dynamic understanding [38]. Finite element analysis predicted tree response to turbulent kinetic energy absorption over time [39]. A novel method for classifying leaf and wood in point clouds was introduced in [40]. Eloy et al. developed MECHATREE, a computational growth model applicable to dicots and conifers [41].

A comparison of the different tree modeling methods is given in Table 1.

**Table 1:** Comparison of different tree modeling methods

Model	Assumptions	Advantages	Limitations
Pipe model	Homogeneity of pipe system, conservation of flow, hierarchy of branches, symmetry, uniform flow, resource limitation	Simplicity, conceptual clarity, hierarchical structure	Simplification, lack of realistic deformation, not suitable for palm family
Classical rod theory	Tree structure as rods, homogeneous rods, uniform flow, conservation of mass and momentum, simplification of tree shape	Flexibility, realistic structural representations, applicability to complex shapes	Neglects some realistic properties, and complexity, not suitable for palm family
Cosserat rod theory	Tree branches and stems are elastic, continuous deformation, no bending resistance, hierarchical structure, gravity as an external force	Torsion deformations, three-dimensional modeling, realistic representations	Data requirements, computational complexity, not suitable for palm family
Biomechanics based model	Environmental Conditions, Resilience and Failure, Structural Heterogeneity	Comprehensive understanding, customization, realistic and holistic	Data and parameterization, complexity and computation, interactions with biological factors, validation, not suitable for palm family
Proposed model	DBH to accommodate the variations in the coconut tree due to the cultivation practices, the coconut tree trunk is taken as the conical frustum	Suitable for the palm family. Consider the effect of wind, tropism, and gravitropism	Not suitable for trees with branches, not tested in other trees in the palm family

The proposed procedure-based growth model incorporates primary and secondary growth factors in coconut trees. Primary growth is modeled using gravitropism, sunlight availability, and wind effects via the cantilever approximation method. Secondary growth considers trunk diameter variation and farming practices like irrigation and fertilization, with DBH (Diameter at Breast Height) as an input parameter. A sensor-equipped unmanned coconut tree climber is used for non-destructive parameter measurements, processed using advanced algorithms. The significant contributions of this research work are.

- Modeling of primary and secondary growth of the coconut tree.
- Development of a coconut tree climber for measuring the coconut tree parameters.
- Method for measuring the coconut tree parameters from the sensor metadata.
- Evaluation of the temporal and spatial growth characteristics of both primary and secondary growth of the coconut tree.

The rest of this paper is arranged as follows. "Materials and methods" section presents the materials and method. “Experiments and results” section explains the experiments and results. "Conclusion" section concludes the paper with the findings of the research work.

## Materials and methods

Due to their branchless trunks, colossal leaf crowns, and greater vulnerability to changes in abiotic conditions, existing growth models for trees are not suitable for the palm family. This paper proposes a procedure-based tree model for coconut trees using the Cosserat rod theory with the influence of abiotic factors.. By integrating biomechanics and growth principles, such a model can provide insights into the structural development of trees and their responses to abiotic factors. Using the Cosserat rod theory, three configurations are defined to explain the growth of coconut trees. Reference, relaxed, and current configurations describe the coconut tree's primary and secondary growth. In the proposed model, three configurations are used to explain the growth of the coconut tree. The reference configuration describes the material introduced to the deformed rod. In the relaxed configuration, external forces are disregarded, whereas these forces are considered in the current configuration. Primary growth considers height, growth direction, and abiotic factors. The diameter variation and influence of abiotic factors are considered in the secondary growth of the coconut tree.

The combination of primary growth (vertical upward growth) and secondary growth (lateral girth increase) leads to the overall development and expansion of the coconut tree. This growth is observed in the increasing height of the tree and the thickening of the trunk. The overall growth is understood by incorporating both primary and secondary growth. The abiotic factors influencing tree growth include sunlight, wind, gravitropism, cultivation practices, soil properties, temperature, humidity, topography, altitude, and elevation.. Coconut trees exhibit monopodial growth and produce leaves and inflorescences from the apex. The trunk elongates as the tree grows, adding new leaves regularly. Two main subsections are presented in this section namely- coconut tree modeling and robot-based coconut tree parameter measurement. The coconut tree modeling subsection explains the coconut tree configurations (reference, relaxed, and current), and primary and secondary growth. The robot-based coconut tree parameter measurement subsection presents the robotic coconut tree climber and coconut tree parameter measurements.

### Coconut tree modeling

**Fig. 1:**
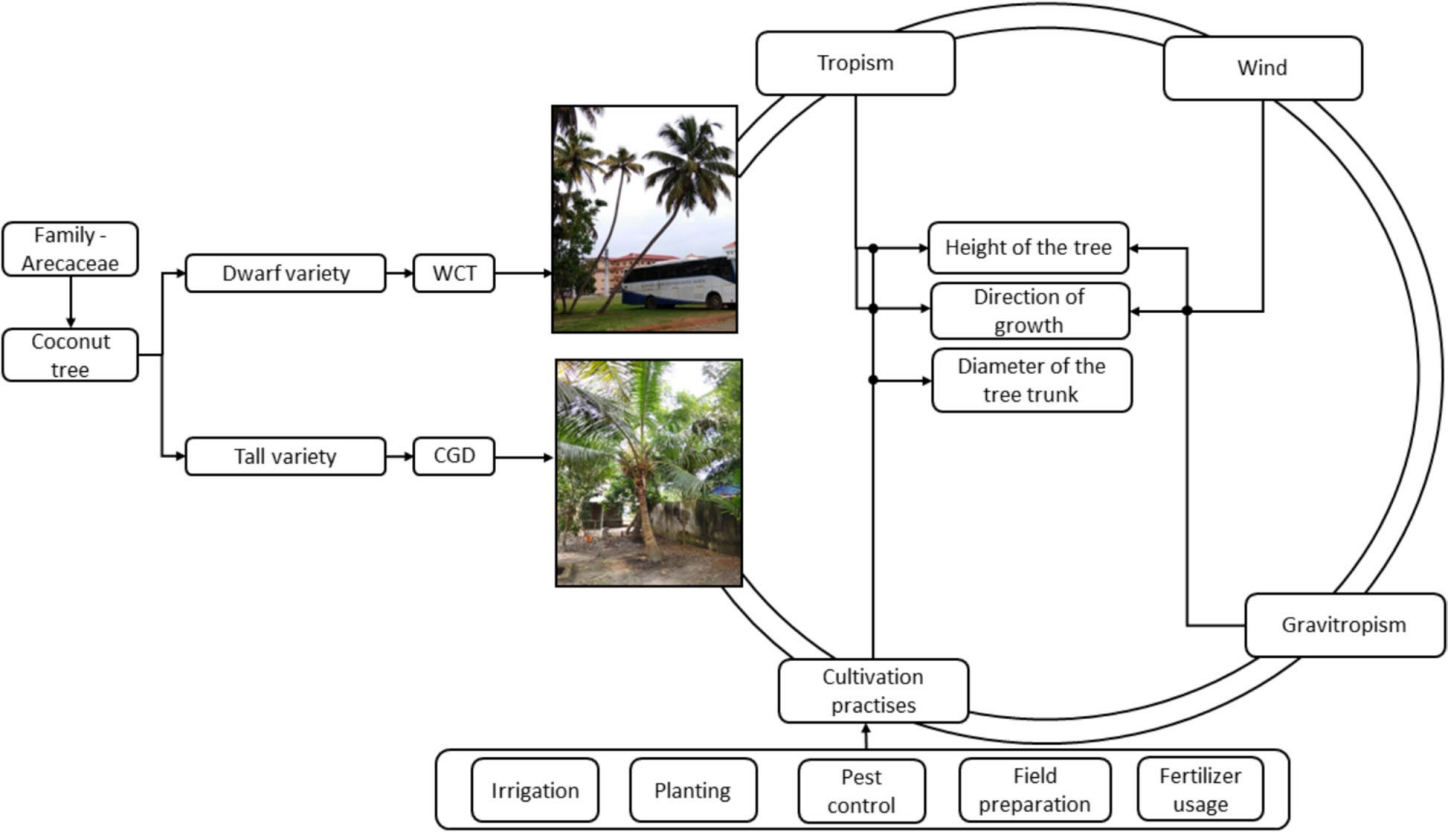
Tree modeling block diagram

The coconut tree, a member of the Arecaceae family, has two main varieties. The West Coast Tall (WCT) coconut tree is a popular cultivar known for its tall stature. It is frequently encountered in the coastal areas of India, specifically on the western coast, which is why it is referred to as WCT. The Chowgat Green Dwarf (CGD) coconut tree is a hybrid cultivar known for its compact size and high productivity. Its dwarf stature characterizes it. The proposed model considers the coconut tree trunk's height, direction of growth, and diameter. As shown in Fig. 1. The height of a coconut tree is influenced by cultivation practices and tropism. Tropism refers to the plant's directional growth response to external stimuli, while phototropism and gravitropism are the growth responses of plants. Coconut trees exhibit positive phototropism towards light sources and negative gravitropism against gravity. The choice of coconut tree varieties and spacing between trees also affect their height. Pruning practices, adequate nutrition, fertilization, and water supply are crucial for healthy growth and development. The direction of growth is impacted by the tropism and wind. Cultivation practices are the abiotic factor that influences the diameter. Tropism and wind significantly influence the growth and direction of coconut trees. Strong winds in coastal regions can cause anemotropism, where trees adapt their growth pattern by leaning or bending towards prevailing winds, reducing wind resistance, and increasing stability. Cultivation practices, including spacing, fertilization, irrigation, pruning, and soil conditions, significantly impact coconut tree trunk diameter, with closer plantings leading to resource competition, wider spacing promoting thicker trunks, and optimal fertilization, irrigation, pruning, and soil management playing essential roles in healthy growth and development. Two assumptions are considered as part of the modeling of the coconut tree 1. Considering the DBH to accommodate the variations in the coconut tree due to the cultivation practices. 2. To determine the geometrical parameters, the coconut tree trunk is taken as the conical frustum.

#### Coconut tree configurations

**Fig. 2:**
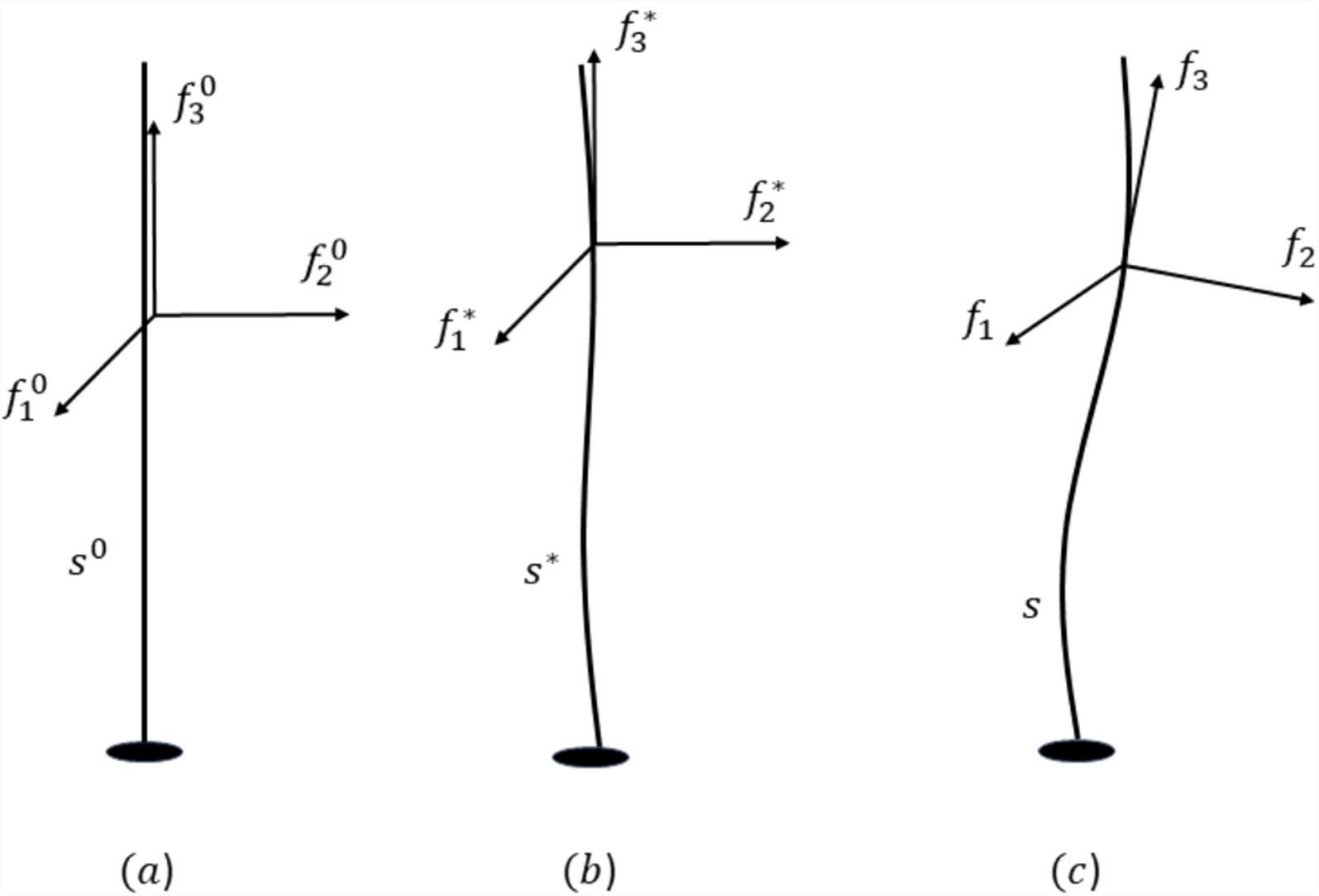
Three configurations of growing rod, **a** reference, **b** relaxed, and **c** current configuration

The proposed model is explained with the help of the three configurations of the growing rods—reference, relaxed, and current as in [42] shown in Fig. 2. Considered all potential material points and time parameter combinations in the reference configuration. The relaxed configuration does not consider the external effects of physical forces. The current configuration gives the cross-section’s exact information, including the physical forces' effects. The configurations are explained separately in the following subsections. In this document, the diacritic ° and * are employed to denote the reference configuration and relaxed configuration, respectively. All the points in the three configurations are considers as the three orthonormal basis function ($${f}_{1}, {f}_{2}, {f}_{3})$$ in the Euclidean space (E).

##### Reference configuration

The reference configuration accommodates adding extra material to the deformed rod. In the reference configuration, the material addition is time-independent, and all the points in the rod are considered in the three-dimensional space [43]. The given equations can express the reference configuration. The basic equations are taken from [44].

**Fig. 3:**
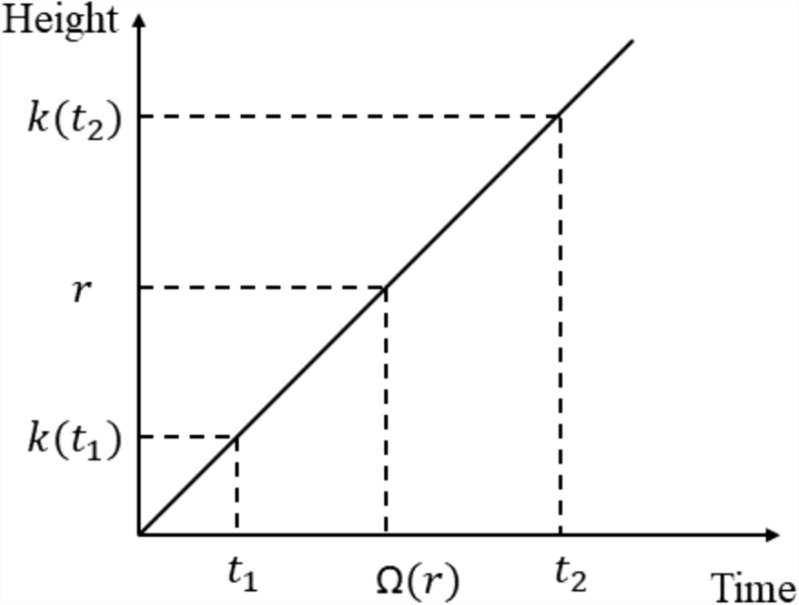
The aggregate height of the coconut tree growth is represented as a linear function

In Fig. 3, two-time values ${t}_{1}$ and ${t}_{2}$ are considered. At the time ${t}_{1}$ the height is taken as k (${t}_{1}$) and at time ${t}_{2}$ the height is taken as k (${t}_{2}$). The inverse function Ω gives the date of appearance of the material parameter (r) located on the base curve of the tree trunk.. All possible points that the rod area can achieve under the curve can be represented mathematically on Eq. (1).

1 $$\left[0,k\left(t\right)\right]\ni r-\to {s}^{0}\left(r\right),{f}_{1}^{0}\left(r\right), {f}_{2}^{0}(r)\in {E}^{3}$$

The primary growth is mentioned by the increase in the length of the base curve represented as $k(t)$. $k(t)$ is always an increasing function of time. The growth velocity ${a}_{v}$ is defined as ${a}_{v}$ = $\frac{dk(t)}{dt}$. The possible material points can be expressed as $A=\{(r,t)|t \in {R}^{+}, r\in (0, k(t))$. The two boundaries are $r=0$ and $r= k(t)$, the origin and the end point of the rod, which is given as $\left(r,t\right)=\left(0,t\right)\in R, \left(r,t\right)=\left(k\left(t\right),t\right) \in A$. At a particular time, the material point (a point in the curve), can be represented as $(r,f\left(r)\right)\in A$.The initial conditions of the strains function $y$ and $w$ are given as ${y}^{0}=(0, {\beta }^{0}, {\gamma }^{0})$ and ${w}^{0}=(0, 0, 1)$ and compared with the Serret Frenet formula, $\frac{d{f}_{i}^{0}}{dr}= {y}^{0}\times {f}_{i}^{0}$ and $\frac{d{s}^{0}}{dr}= {f}_{3}^{0}$. The reference configuration represents the growth in the longitudinal direction, and it depends on the initial curvature (${\beta }^{0}$) and initial torsional strain( ${\gamma }^{0})$. The biotic and abiotic factors alter the growth of the tree. The main trigger for the mechanical stimuli in plants are wind and self-weight. The gradual growth of the tree is considered as the deposition of new materials on the existing material. With the newly deposited material, the self-weight, and the effect due to gravity increase. In addition to this, the tree's geometry also changes with respect to the material addition. Because of these rapid mechanical responses, the tree trunk or stem become more thicker to resist the breaking [45].

##### Relaxed configuration

Any tree's growth involves three processes: growth, shape change, and material property change [46]. Compared to the current configuration relaxed configuration is not considering the external forces. Primary growth, secondary growth and tropism are the three major factors that make change to the relaxed configuration. The relaxed configuration is represented by the following equation (Eq. (2)).

2 $$A\ni (r,t)-\to {s}^{*}\left(r,t\right), {{f}_{1}}^{*}\left(r,t\right), {{f}_{2}}^{*}(r,t)\in {E}^{3}$$

In Eq. (2),${s}^{*}\left(r,t\right)$ represent the positional variation and the ${{f}_{1}}^{*}\left(r,t\right), {{f}_{2}}^{*}(r,t)$ represents the orientation of the rod. The third function can be obtained from the other two, ${{f}_{3}}^{*}={ {f}_{1}}^{*}\times {{f}_{2}}^{*}$. So, to represent the orientational information ${{f}_{1}}^{*}\left(r,t\right)$,and ${{f}_{2}}^{*}(r,t)$ are required. The ${{f}_{i}}^{*}$ ($i=\text{1,2},3$) is given as the director of the rod growth. The strain vector valued function as in [47] is given as ${\partial }_{r}{{f}_{i}}^{*}={y}^{*}\times {{f}_{i}}^{*}$ and ${\partial }_{s}r={w}^{*}$. The components of $y$ and $w$ with respect to ${{({f}_{i})}^{*}}_{i=\text{1,2},3}$, ${y}^{*}=\left({{y}_{1}}^{*}, {{y}_{2}}^{*},{{y}_{3}}^{*}\right)$ and ${w}^{*}=\left({{w}_{1}}^{*}, {{w}_{2}}^{*},{{w}_{3}}^{*}\right)$.

##### Current configuration

The current configuration includes the effect of the physical forces. The current configuration can be represented by the following equation Eq. (3).

3 $$A\ni (r,t)-\to s\left(r,t\right), {f}_{1}\left(r,t\right), {f}_{2}(r,t)\in {E}^{3}$$

The representation of orientation, strain-valued functions, and growth directors is identical to that of a relaxed configuration. During this derivation the rod is considered as fixed at the origin. So, the boundary conditions are ${f}_{i}\left(0,t\right)$ = ${f}_{0i}$ and $s\left(0,t\right)$ = $0$. The main mechanical stimuli to cause the thigmomorphogenesis are the abiotic factors include gravity, sunlight, and wind. Due to self-weight inclination and direction of growth changes which results in differential growth. The tropisms can be divided into two namely gravitropism, and tropism, effect due to sunlight. Gravitropism is due to the self-weight of tree which influences all the responses of the tree caused by any mechanical stimuli. For example, due to wind a mechanical stimulus is generated but the response of the tree will be the addition of the effect due to its self-weight. Tropism is due to sunlight.

#### Primary growth with the influence of abiotic factors

The monocots, like coconut trees, have abundant vascular bundles inside the ground tissue. The secondary growth is not significant compared to primary growth. The growth in length of the tree is called apical growth. The coconut trees are showing continuous apical growth. The secondary growth, the thickening of the tree trunk is much less in Coconut trees than primary growth. The coconut tree cortex is same as that of coniferous trees, the growth stress to the inner bark and the absolute strain is inversely proportional to the height [48]. The base curve is formed by the initial strains, the deciding factor for the orientation of the tree. The primary growth is mainly influenced by the phototropism, gravitropism, and effect of the self-weight. The direction of movement depends on the sunlight availability, gravity, and the wind speed. The curvature ${\beta }^{0}$ and torsion ${\gamma }^{0}$ can be represented as in [49] (Eqs. 4 and 5).

4 $${\gamma }^{0}\left(r\right)= \widehat{{\gamma }^{0}}(r,f\left(r\right),s\left(r, f\left(r\right)\right), {f}_{i} \left(r,f\left(r\right)\right)\dots \dots )$$

5 $${\beta }^{0}\left(r\right)= \widehat{{\beta }^{0}}(r,f\left(r\right),s\left(r, f\left(r\right)\right), {f}_{i} \left(r,f\left(r\right)\right)\dots \dots )$$

If no external influence, the ${\beta }^{0}={\gamma }^{0}=0$. The base curve become straight line and the orientation can be expressed by the ${f}_{03}$. Consider a parameter to represent the favourable growth direction, ${f}_{p}$. Initial position is considered as ${f}_{3}(r,f(r))$, assuming that after the increase in length $\Delta$, ${f}_{3}(r,f(r))$ is reaches the favourable growth direction where ${f}_{3}\left(r+\Delta , f\left(r\right)\right)= {f}_{p}$. Using Taylor’s approximation as shown in Eq. (6)

$${f}_{3}\left(r+\Delta ,f\left(r\right)\right)= {f}_{3}\left(r,f\left(r\right)\right)+\Delta {\partial }_{r}{f}_{3}\left(r,f\left(r\right)\right)+\frac{{\Delta }^{2}}{2}{\partial }_{rr}(r,f(r))$$

6 $$={(\beta }^{0}\left(r\right)\Delta +\frac{d{\beta }^{0}}{dr}(r)\frac{{\Delta }^{2}}{2}){f}_{1}(r,f(r))+{\beta }^{0}(r){\gamma }^{0}(r)\frac{{\Delta }^{2}}{2}{f}_{2}(r, f(r))+(1-{{\beta }^{0}\left(s\right)}^{2}\frac{{\Delta }^{2}}{2}){f}_{3}(r,f(r))$$

Taking the scalar product with the direction vectors ${f}_{i}(r,f(r))$, where gives the projection of the desired direction over the direction vectors and obtaining solutions of ${\beta }^{0}$, $\frac{d{\beta }^{0}}{d\gamma }$, ${\gamma }^{0}$ Eq. (7)–(12).

7 $${f}_{p}.{f}_{1}\left(r,f\left(r\right)\right)= {\beta }^{0}\left(r\right)\Delta +\frac{d{\beta }^{0}}{dr}(s)\frac{{\Delta }^{2}}{2}$$

8 $${f}_{p}.{f}_{2}\left(r,f\left(r\right)\right)= {\beta }^{0}\left(r\right){r}^{0}\frac{{\Delta }^{2}}{2}$$

9 $${f}_{p}.{f}_{3}\left(r,f\left(r\right)\right)= {1-\beta }^{0}{(r)}^{2}\frac{{\Delta }^{2}}{2}$$

Then the solutions for the ${\beta }^{0}$, $\frac{d{\beta }^{0}}{d\gamma }$, ${\gamma }^{0}$

10 $${\beta }^{0}\left(s\right)=sgn \left({f}_{p}.{f}_{1}\left(r,f\left(r\right)\right)\right)\frac{{\beta }_{max}}{2}\sqrt{2(1-{f}_{p}.{f}_{3}(r, f\left(r\right))}$$

11 $$\frac{d{\beta }^{0}(s)}{d\gamma }= \frac{{{\beta }^{2}}_{max}}{2}\left({f}_{p}{f}_{1}\left(r,f\left(r\right)\right)-sgn \left({f}_{p}{f}_{1}\left(r.f\left(r\right)\right)\right)\sqrt{2(1-{f}_{p}{f}_{3}(r,f(r))}\right)$$

12 $${\gamma }^{0}\left(r\right) = \left\{\begin{array}{c} sgn ({f}_{p}{f}_{1}\left(r,f\left(r\right)\right){\beta }_{max}\frac{{f}_{p}{f}_{2}\left(r,f\left(r\right)\right)}{\sqrt{2(1-{f}_{p}{f}_{3}(r,f(r))}} if{\beta }^{0}\left(r\right) \ne 0 \\ 0, if {\beta }^{0}\left(r\right) = 0\end{array}\right.$$

Next subsection discussed the effect of sunlight and wind in the primary growth to modify the equation.

##### Effect of sunlight

The trees respond to light depending on different factors like climatic sonditions, type of tree, altitude, and cultivation practises [50]. The Total Available Sunlight (TAS) can be represented as a sum of Overall Mean Sunlight (OMS), Percentage of Full Sunlight(PFS), Variation of Sunlight with respect to height (VS(H)) and an error (E) (Eq. (13).

13 $$TAS=OMS+PFS+VS\left(H\right)+E$$

The final angle of deflection due to sunlight and wind is represented as ${\theta }_{p}^{f}$, given in Eq. (14).

14 $${\theta }_{p}^{f}= \theta +{\theta }_{S}+{\theta }_{w}$$

The coconut tree grows in a direction in which the sunlight intensity is high. The presence of shade or lack of sunlight leads to the shade avoidance response. In which tree grows in the direction to get maximum sunlight. As a result of this the stem of the tree become taller and slender. If this is occurring in a dense canopy of vegetation, there is a little variation because the coconut trees are vying for life, and as a result, the mechanical reaction is reduced since the extension of the stem might damage the tree.

##### Effect of wind

One of the abiotic factors that influences the growth direction of coconut tree is wind. The preferential growth direction is changed because of the sunlight and the wind. The dense arrangement of coconut trees in this field exhibits minimal wind impact, as neighboring trees serve as a natural shield, effectively distributing the effect among them. In open habitat Coconut trees undergo drastic changes in the direction of growth, bend in the tree trunk due to wind. The kinematics of the tree growth also change because of wind. In some cases, the wind can change the orientation of the trees [51]. The sway of the coconut trees or the effect due to the wind depends on the frequency of the wind. If the frequency is high the mechanical stimuli lead to morphogenetic signals [52]. With the help of linear elastic constitutive relation, the curvature can be represented as in the Eqs. (15 and 16).

15 $$\beta \left(r,t\right)= {\beta }^{*}\left(r,t\right)+\frac{q(r,t)}{YI(r,t)}$$

16 $$\beta \left(r,t\right)= {\beta }^{*}\left(r,t\right)+\frac{10 q\left(r,t\right)[x\left(r,t\right)-x\left(0,t\right)]}{3{Y}^{2}{\rho }^{\prime}\pi y\left(r,t\right) {[x\left(r,t\right)}^{5}-{x\left(0,t\right)}^{4}x\left(r,t\right)+{x\left(0,t\right)}^{5}]}$$

**Fig. 4:**
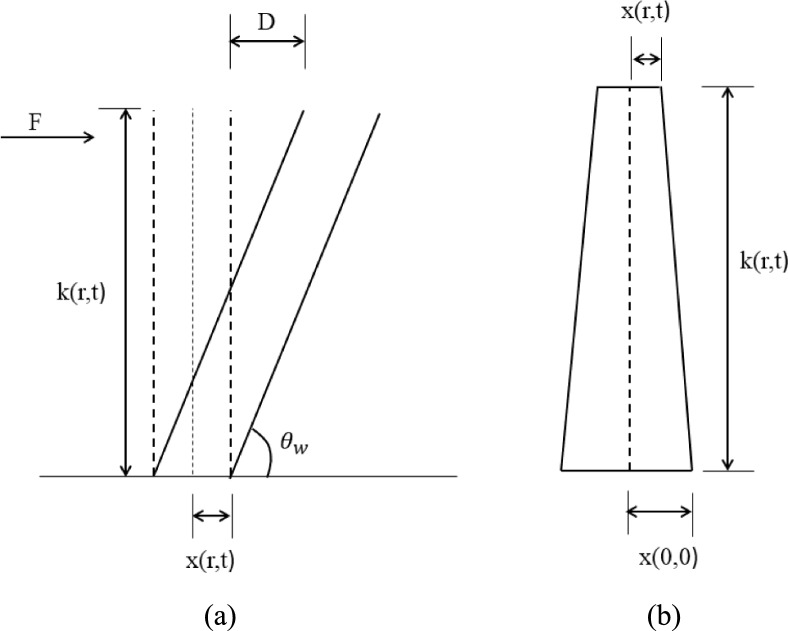
**a** Consider the coconut tree trunk as a conical frustum, **b** Cantiliver model to explain the effect of wind

The curvature of the coconut tree is inversely proportional to bending moment. The bending moment caused by the wind is calculated using the cantilever model. The bending moment is proportional to the second derivative of the beam deflection with respect to it’s initial position (with respect to the Y axis) (of the beam (as shown in Fig. 4a) [53] Then the bending moment is given as ${B}^{\prime}=YI\frac{{\partial }^{2}D}{{\partial k}^{2}\left(r,t\right)}$.

The tree trunk deflection is given by $D=\frac{F{k(r,t)}^{3}}{3YI}$ and t ${\theta }_{w}={cos}^{-1}(\frac{k\left(r,t\right)}{D+x\left(r,t\right)})$ respectively. Assume that the coconut tree trunk is a conical frustum in order to determine its geometrical values. (as shown in Fig. 4b) with bottom radius as $x(\text{0,0})$ and top radius at height $k(r,t)$ is represented as $x(r,t)$. The moment of inertia and diameter of the trees are represented as in Eq. (17) & Eq. (18)

17 $$I = { }\frac{{\rho^{\prime}\pi k\left( {r,t} \right)}}{10}[\frac{{x\left( {r,t} \right)^{5} - x\left( {0,t} \right)^{4} x\left( {r,t} \right) + x\left( {0,t} \right)^{5} }}{{x\left( {r,t} \right) - x\left( {0,t} \right)}}]$$

18 $$\nabla =\frac{F{k(r,t)}^{3}}{\frac{{3Y\rho }^{\prime}\pi k\left(r,t\right)}{10}[\frac{{x\left(r,t\right)}^{5}-{x\left(0,t\right)}^{4}x\left(r,t\right)+{x(0,t)}^{5}}{x\left(r,t\right)-x\left(0,t\right)}]}$$

Coconut trees face various types of physical stress, with wind loading being the primary and most influential form of stress they must endure. The wind can come swiftly, intermittently, and as a dynamic event. The wind is making great impact on individual trees compared to the trees growing in the deep forest. The effect of the wind forces become more critical with the age, height, and number of branches. In the case of the coconut trees, their slender structure without branches and the big crown of leaves in the treetop make them more sensitive to the wind force.

##### Apical growth

The change of curvature due to the sunlight and wind is represented as $p=\frac{2{v}^{\gamma }}{{s}^{2}}\alpha \text{sin}({\theta }_{p}^{f}-\theta )$ with $\alpha$ as the maximum differential maturation strain. To calculate the exact solution of the small defection rate of change of the contact couple and position with respect to material is claculated, which is given as${\partial }_{r}q=-{p}_{y}cos\theta$, ${\partial }_{t}{\beta }^{*}= \frac{{\partial }_{t}YIq}{{(YI)}^{2}}$, ${\partial }_{r}\theta = {\beta }^{*}+ \frac{q}{YI}$ and ${\partial }_{r}s= {cos\theta }_{0}i+(sin{\theta }_{0}+\left(\theta -{\theta }_{0}\right)cos{\theta }_{0})j$ with initial conditions$q\left(k\left(t\right), t\right)=0$,${\beta }^{*}\left(r,f\left(r\right)\right)= {\beta }_{max}\text{sin}(\frac{{\theta }_{p}^{f}-{\theta }_{0}}{2})$,$\theta \left(0,t\right)= {\theta }_{0}$,$s\left(0,t\right)=0$.

**Fig. 5:**
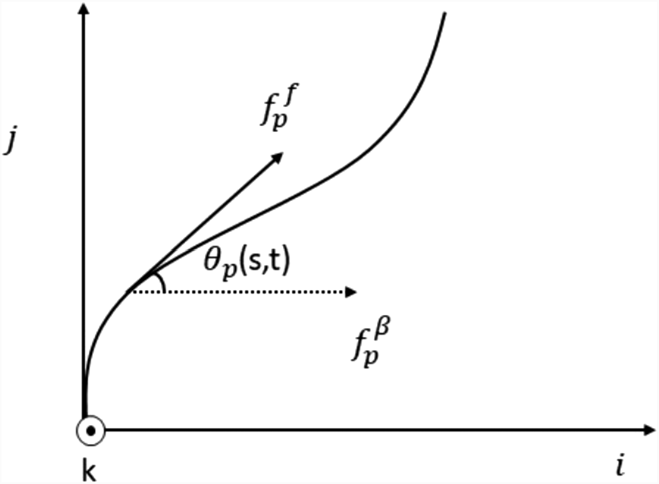
The movement of the coconut tree trunk subjected to wind and sunlight

The normalized vector normal to the preferential growth direction ${f}_{p}$ considering the growth and gravitropism and preferential growth direction including the effect of sunlight and wind ${{f}_{p}}^{*}$ (${f}_{p}^{\gamma })$ is obtained as ${f}_{p}^{\gamma }= \frac{{f}_{p} \times {{f}_{p}}^{*}}{\Vert {f}_{p} \times {{f}_{p}}^{*}\Vert }$ and the vector perpendicular to that vector ${{f}_{p}}^{\beta }= {f}_{p}^{\gamma }\times {{f}_{p}}^{*}$ as shown in Fig. 5. The movement of the tree trunk because of sunlight and wind is represented as ${\theta }_{p} (s,t)$. Then the vector to represent the direction of growth of tree. The preferential growth direction considering the effect of wind and sunlight is given as (Eq. (19)) (In this section $^{\prime}\times ^{\prime}$ represent the cross product of vectors)

19 $${{f}_{p}}^{F}= {f}_{p}\text{cos}\theta \times {{f}_{p}}^{\beta }\text{sin}\theta$$

Then the initial position (The direction of ${f}_{p}$) is considered as ${f}_{3}(r,f(r))$, assuming that after increase in length $\Delta$, the current position is reaching the favourable growth direction, given as (Eq, (20)).

20 $${f}_{3}\left(r+\Delta , f\left(r\right)\right)= {{f}_{p}}^{F}$$

Using Taylor’s approximation

$${f}_{3}\left(r+\Delta ,f\left(r\right)\right)= {f}_{3}\left(r,f\left(r\right)\right)+\Delta {\partial }_{r}{f}_{3}\left(r,f\left(r\right)\right)+\frac{{\Delta }^{2}}{2}{\partial }_{rr}(r,f(r))$$

21 $$={\beta }^{0}\left(r\right)\Delta +\frac{d{\beta }^{0}}{dr}(r)\frac{{\Delta }^{2}}{2}){f}_{1}(r,f(r))+{\beta .}^{0}(r){\gamma }^{0}(r)\frac{{\Delta }^{2}}{2}{f}_{2}(r, f(r)+(1-{{\beta }^{0}\left(s\right)}^{2}\frac{{\Delta }^{2}}{2}){f}_{3}(r,f(r))$$

Taking the scalar product with the direction vectors${f}_{i}(r,f(r))$, this is giving the projection of the desired direction over the direction vectors to measure how much of the preferential growth direction vector in the direction of${f}_{i}(r,f(r))$. It calculates the component of the preferential growth vector that aligns with directional vectors (Eqs. (22, 24).

22 $${{f}_{p}}^{F}.{f}_{1}\left(r,f\left(r\right)\right)= {\beta }^{0}\left(r\right)\Delta +\frac{d{\beta }^{0}}{dr}(s)\frac{{\Delta }^{2}}{2}$$

23 $${{f}_{p}}^{F}.{f}_{2}\left(r,f\left(r\right)\right)= {\beta }^{0}\left(r\right){r}^{0}\frac{{\Delta }^{2}}{2}$$

24 $${{f}_{p}}^{F}.{f}_{3}\left(r,f\left(r\right)\right)= {1-\beta }^{0}{(r)}^{2}\frac{{\Delta }^{2}}{2}$$

The curvature of the coconut tree concerning the path (${\beta }^{0}$), the rate of change curvature with respect material $\frac{d{\beta }^{0}}{d\gamma }$ and the torsion position (${\gamma }^{0}$) are given below (Eqs. 25, 26, 27)).

25 $${\beta }^{0}\left(s\right)=sgn \left({{f}_{p}}^{F}.{f}_{1}\left(r,f\left(r\right)\right)\right)\frac{{\beta }_{max}}{2}\sqrt{2(1-{{f}_{p}}^{F}.{f}_{3}\left(r, f\left(r\right)\right))}$$

26 $$\frac{d{\beta }^{0}(s)}{d\gamma }= \frac{{{\beta }^{2}}_{max}}{2}\left({{f}_{p}}^{F}{f}_{1}\left(r,f\left(r\right)\right)-sgn \left({{f}_{p}}^{F}{f}_{1}\left(r.f\left(r\right)\right)\right)\sqrt{2(1-{{f}_{p}}^{F}{f}_{3}(r,f(r))}\right)$$

27 $${\gamma }^{0}\left(r\right) = \left\{\begin{array}{c} sgn ({{f}_{p}}^{F}{f}_{1}\left(r,f\left(r\right)\right){\beta }_{max}\frac{{{f}_{p}}^{F}{f}_{2}\left(r,f\left(r\right)\right)}{\sqrt{2(1-{{f}_{p}}^{F}{f}_{3}(r,f(r))}} if{\beta }^{0}\left(r\right) \ne 0 \\ 0, if {\beta }^{0}\left(r\right) = 0\end{array}\right.$$

#### Secondary growth with the influence of abiotic factors

Compared to primary growth the secondary growth is minimum in coconut trees (palm family) and are different from the dicots and gymnosperms. The coconut trees are showing diffused secondary growth that is different from normal secondary growth due to the division of the xylem cells inside the meristem. In the diffused secondary growth, the radius of the tree trunk is varying because of the split and expansion of parenchyma cells. In general, cultivation practices that promote healthy tree growth, such as adequate nutrient supply, water management, and pest control, can contribute to an increase in DBH over time. Proper cultivation practices can provide favourable conditions for trees to develop a robust root system, efficient photosynthesis, and overall structural growth. The rate of change of the radius is mentioned as the radial growth velocity $${V}^{r}= {\partial }_{t}x\left(r,t\right), {x}_{0} >0$$ and the area$${A}_{r}\left(r,t\right)= \pi {x}^{2}(r,t)$$,$${A}_{r}(r,t)\in A$$. The areal mass density is represented as $${\rho }^{{\prime}{\prime}{\prime}}= {\rho }^{\prime}{A}_{r}\left(r,t\right)= {\rho }^{\prime} \pi {x}^{2}(r,t)$$ and the bulk modulus as $$B = \frac{{dp^{\prime}}}{{{\raise0.7ex\hbox{${dv^{\prime}}$} \!\mathord{\left/ {\vphantom {{dv^{\prime}} {v^{0} }}}\right.\kern-0pt} \!\lower0.7ex\hbox{${v^{0} }$}}}}$$. The negative sign indicates that the increase in pressure is accompanied by the decrease in volume. After integrating and rearranging the above equation of the Bulk modulus, $${c}^{\prime}$$ represents the integrating constant added during the integration process.

28 $${A}_{r}\left(r,t\right)= \frac{-{\rho }^{\prime}g}{B}{c}^{\prime}{e}^{ \frac{-{\rho }^{\prime}g}{B}k\left(r,t\right)}$$

Total area of the coconut tree trunk can be expressed as (Eq. (29))

29 $$\sum \pi {x(r,t)}^{2}= \frac{-{\rho }^{\prime}g}{B}{c}^{\prime}{e}^{ \frac{-{\rho }^{\prime}g}{B}k\left(r,t\right)}$$

Considering a cross section at a particular $k(r,t)$, the radius at that point can be expressed as (Eq. (30))

30 $$x\left(r,t\right)= \sqrt{\frac{-{\rho }^{\prime}g}{B\pi }{c}^{\prime}{e}^{ \frac{-{\rho }^{\prime}g}{B}k(r,t)}}$$

From Eq. (30) we can obtain the base radius as

31 $$x\left(0,t\right)= \sqrt{\frac{-{\rho }^{\prime}g}{B\pi }{c}^{\prime}}$$

From Eq. (30) we can obtain the radius at a height of 1.4 m from ground (half of the DBH) as

32 $$x\left(1.4,t\right)= \sqrt{{c}^{\prime}{e}^{ \frac{-{\rho }^{\prime}g}{B}k(1.4,t)}}$$

By differentiating Eq. (30) we got the radial growth velocity as

33 $${V}^{r}= \frac{\frac{({{\rho }^{\prime}g)}^{2}}{{B}^{2}\pi }{c}^{\prime}{e}^{ \frac{-{\rho }^{\prime}g}{B}k(r,t)}{a}_{v}}{2\sqrt{\frac{-{\rho }^{\prime}g}{B\pi }{c}^{\prime}{e}^{ \frac{-{\rho }^{\prime}g}{B}k(r,t)}}}$$

Substitute ${e}^{ \frac{-{\rho }^{\prime}g}{B}k(r,t)}=\Gamma$, and $\frac{{-\rho }^{\prime}g}{B\pi }{c}^{\prime}= \zeta$ then Eq. (30) becomes.

34 $${V}^{r}= \frac{{-\rho }^{\prime}g\zeta\Gamma {a}_{v}}{2\sqrt{\zeta\Gamma }}$$

If the apical growth velocity and radial growth velocity are constant, then the length of the tree trunk with respect to time is $k\left(t\right)= {a}_{v}t$. The appearance of the material with respect to time $f(r)=\frac{r}{ {a}_{v}}$ and the material cross-section atr and at time t, $s\left(r,t\right)= {V}^{r}t- \frac{{V}^{r}}{{a}_{v}}s+{s}_{0}$. The strain rate is related to the DMS effect and depends on the ${V}^{r}$ if we consider the contact force and couple. There are differences between the WCT and CGD coconut varieties in terms of ${V}^{r}$, ${A}_{r}\left(r,t\right)$, ${\rho }^{{\prime}{\prime}{\prime}}$ [54].

### Robot based coconut tree parameter measurement.

#### Robotic coconut tree climber

**Fig. 6:**
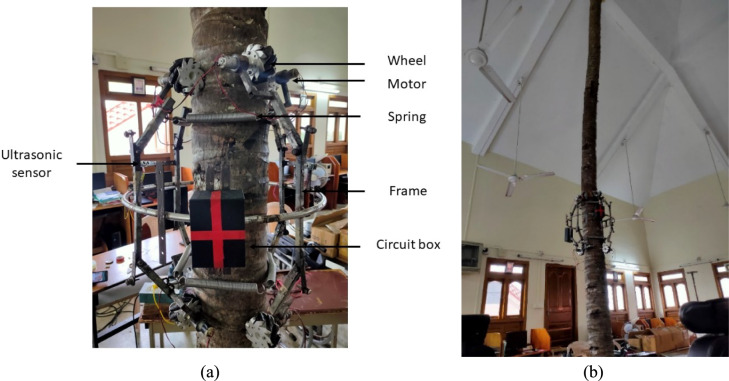
**a** Robotic coconut tree climber (**b**) Lab setup to calibrate the sensor setup

An eight wheeled robot (as shown in Fig. 6a) is built to climb the coconut tree and measure the coconut tree parameter [55]. The robotic coconut tree climber incorporates a spring mechanism that enhances traction on the coconut tree trunk while accommodating variations in trunk diameter. Semicircular rings enable swift and convenient attachment and detachment of the system around the trunk. The climber body is designed to accommodate a robotic arm (RA) and an embedded system controller, which includes a microcontroller unit and motor drivers. The embedded system controller is securely attached to the semicircular ring.The power management block efficiently distributes power to both the climber and the RA, commonly referred to as the robotic harvester. A 5.2 Ah Lithium polymer (LiPo) battery powers the entire system. The RA is equipped with a cutter that is remotely controlled from a ground station via AC power. Additionally, the Emergency Handling System (EHS) is powered by an independent LiPo battery to ensure a safe descent of the climber in cases of complete primary battery discharge or connectivity loss with the ground station via the wireless Bluetooth interface in Amaran.

This battery-operated tree climber is also integrated with a sensor system to measure essential tree parameters. It features two ultrasonic sensors and a 9-axis IMU. The ultrasonic sensors, positioned on the climber’s frames, detect variations in tree trunk diameter. The circuit box houses the controller, battery, Bluetooth module, and a storage unit. During the robot's ascent and descent, sensor data is logged in the storage unit. The Bluetooth-enabled robot is operated via a mobile application, ensuring remote control and monitoring capabilities.

The sensor data collected is processed as in the Fig. 8 and get the tree parameters accurately.

Two ultrasonic sensors are placed on the frames of coconut tree climber to measure the diameter. Each ultrasonic sensor gives the distance between the coconut tree trunk with respect to the frame. IMU data is used to measure the height and direction of growth. IMU and ultrasonic sensors are calibrated in the lab setup (as shown in the Fig 6b) before the real time data measurement. The method to calculate the coconut tree parameters from the sensor metadata is also examined during the lab test.

#### Coconut tree parameter measurement

**Fig. 7:**
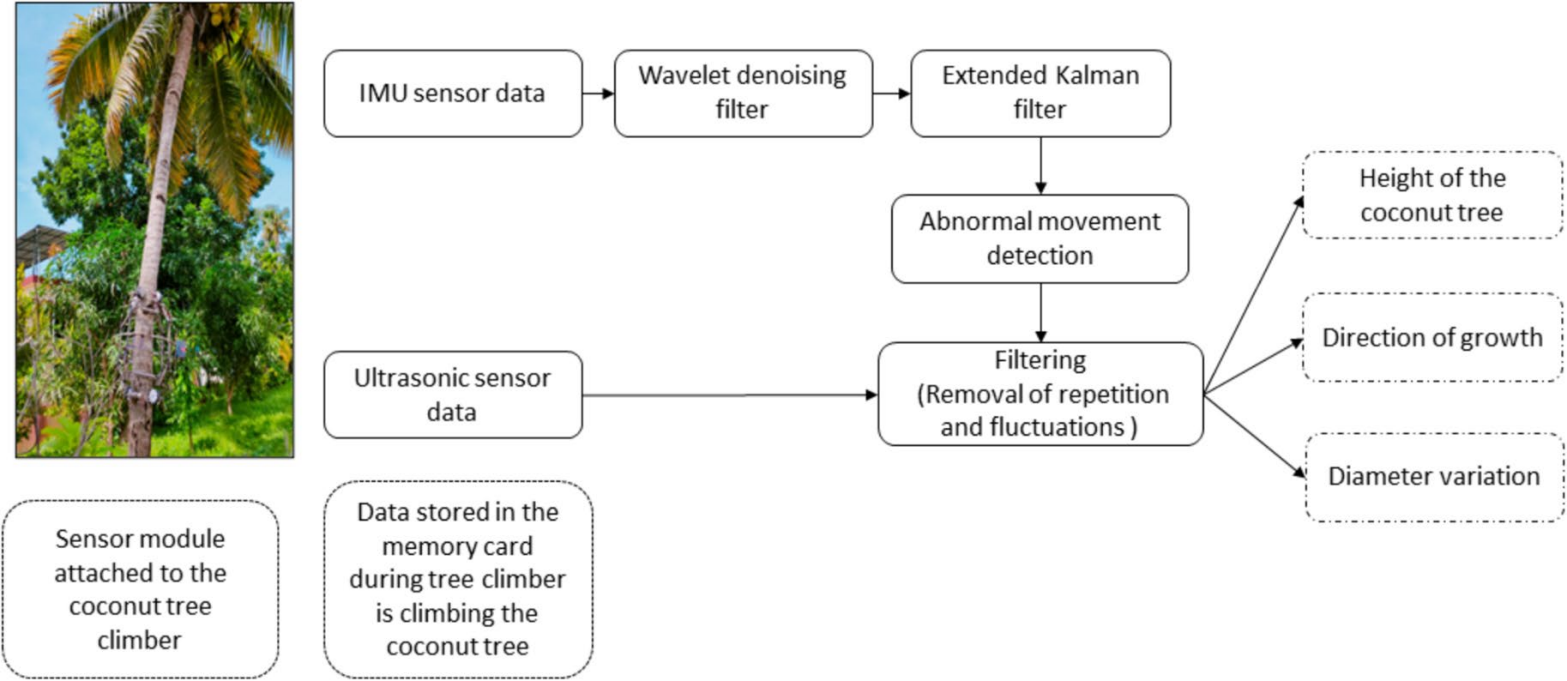
Block diagram–the method used to obtain the coconut tree parameters from the sensor data

The parameters of the coconut tree are extracted from the sensor metadata using a variety of processes, as shown in Fig. 7. The data acquisition process involved obtaining nine-axis IMU sensor data, comprising accelerometer, gyroscope, and magnetometer readings on three axes. To mitigate noise in the sensor data, wavelet denoising was employed using the Daubechies wavelet type and Stein's Unbiased Risk Estimate (SURE) threshold in the time-based wavelet domain. Standard deviation was computed using the median absolute deviation. The denoised signal was reconstructed through the Inverse Discrete Wavelet Transform (IDWT). Subsequently, sensor fusion was conducted using the Extended Kalman Filter (EKF), with a polynomial degree 3 state transition function and a diagonal matrix initialized with variance values for position and orientation in the state covariance matrix. Similarly, the measurement covariance matrix comprised a diagonal matrix with variance values for the measurement noise used in sensor fusion. The EKF outputted position and heading angle data, enabling the detection of movement abnormalities stemming from slip, scars, wedges in tree trunk surfaces, mechanical alignment issues, and the robot's orientation due to the tree structure. Following position filtering, the Y axis denoted height, the X axis represented horizontal shift, and the heading angle indicated the direction of growth, with all values steadily increasing over time. Additionally, two ultrasonic sensors, placed on both sides of the climber frame (as depicted in Fig. 6a, were utilized to measure diameter variations continuously during climbing. Diameter measurement abnormalities, attributed to slips, scars, wedges in tree trunk surfaces, mechanical alignment, and robot orientation due to the tree structure, were rectified through filtering to ensure accurate diameter variations.

##  Experiments and results

The temporal and spatial growth characteristics predicted by the proposed model are compared to Antman's classic rod theory (already cited this work as [44]) and Thomas Guillon's biomechanical growth model (already cited this work as [42]) as part of the evaluation process. The real time temporal growth characteristics of two major varieties of coconut tree, West Coast Tall (WCT) and Chowghat Green Dwarf (CGD) are provided by Central Plantation Crops Research Institute (CPCRI), Kayamkulam, Kerala, India, to evaluate the spatial growth characteristics, growth traits obtained using the coconut tree parameters measured by robotic coconut tree climber are used. The spatial growth traits obtained from the sensor data is compared with the growth characteristics predicted by the proposed model, classical rod theory, and biomechanical growth model. The following parameter values are used to perform the experiments ${\rho }^{\prime}$ = 600 kg/m3, $g$ = 9.8 N/kg, $Y$ = 1.14*10^10 Pa, ${\theta }_{0}$ = 0 rad, ${a}_{v}$ = 0.46 m/y, ${V}^{r}$ = 0.01 m/y, ${s}_{0}$= 0.01 m, $s$= 0.01 m, $\alpha$ = 0.001 m/m, ${\theta }_{p}$ = π/2 rad, ${\beta }_{max}$ = 0 1/m, ${\theta }_{p}^{f}$ = 0.61π rad. We derive the physical and mechanical parameters from references [55] and [56, and we calculated radial and apical growth velocities using data provided by the CPCRI. The preferred direction of growth, both considering and not considering the physical factors, is determined through a trial-and-error method.

### Temporal growth characteristics

The temporal growth characteristics of a tree refer to the patterns and changes in its growth over time. This includes annual growth rings, growth rate, height increment, crown development etc. Real time temporal data of average height variation of the WCT and CGD varieties over a period of 40 years are used for this evaluation.

#### Primary growth

**Fig. 8:**
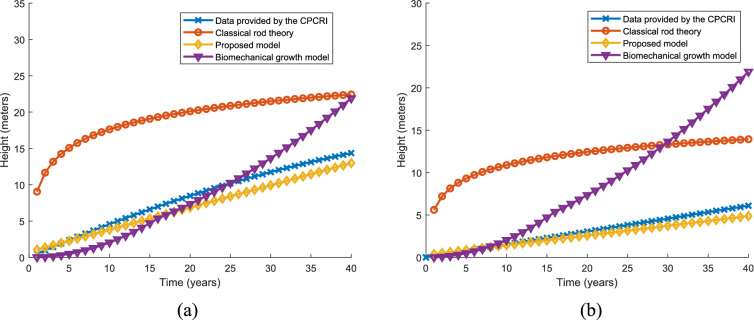
Comparison of the temporal primary growth characteristics (height) predicted by the proposed model for (**a**) WCT coconut palm (**b**) CGD coconut palm

In this section, the temporal primary growth (height variation) predicted by the proposed model is compared with real time data, classical rod theory and biomechanics growth model. In Fig. 8a. variation of height over a period of 40 years is compared with the predicted height by different models for WCT coconut variety. The biomechanical growth model predicted the exponential height variation for WCT coconut variety. The variance is denoted in square meters. The temporal primary growth characteristics predicted by the proposed model shows a variance of 38.7 with respect to the real-time data. Classical rod theory shows a variance of 797 and biomechanical growth model shows a variance of 98.3 with the real time growth characteristic of the WCT. Figure 8b shows the variation of height over 40 years is compared with the predicted height by different models for CGD coconut variety. The height variation predicted by the proposed model shows the better variance of 29.02 with the real time data. Biomechanics growth model shows a variance of 67.8 and Classical rod theory shows a variance of 557.9 with the real time growth characteristic of the WCT.

#### Secondary growth

**Fig. 9:**
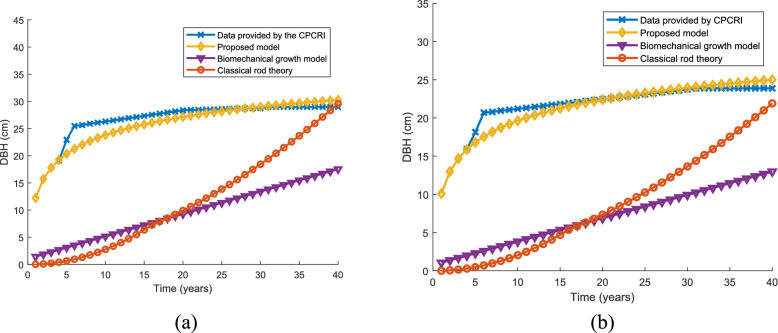
Comparison of the temporal secondary growth (variation of DBH) characteristics predicted by the proposed model for (**a**) WCT coconut palm (**b**) CGD coconut palm

The temporal secondary growth of WCT and CGD predicted by the proposed model is compared with the real time data and two other models—classical rod theory and biomechanics growth model. The DBH variation over a time of 40 years is used for comparison. In general, the WCT variety tends to have a larger DBH compared to the CGD coconut tree. CGD coconut trees have a shorter height and relatively smaller DBH compared to WCT coconut trees. It's important to consider that there can be variations within each variety depending on specific factors and local conditions. In Fig. 9a, the variation of DBH of WCT variety over time predicted by the proposed model and two other models are compared with the real time data. To compare, the variance of the predicted growth by different models with real data are calculated. The variance is denoted in square centimetres. Biomechanical growth model shows maximum variance of 732 and classical rod theory shows variance of 473.4. The proposed model shows better variance compared with real time data, 25.3. In the case of CGD (as shown in Fig. 9b) variety the variances with real time data are 18.3, 278.8, 457.9 for proposed model, classical rod theory and biomechanical growth model respectively. It is very clear from the characteristics shown in Fig. 9a, b and the measured variance, the temporal growth characteristics predicted by the classical rod theory and biomechanical growth model are not suitable for coconut tree modeling.

### Spatial growth

**Fig. 10:**
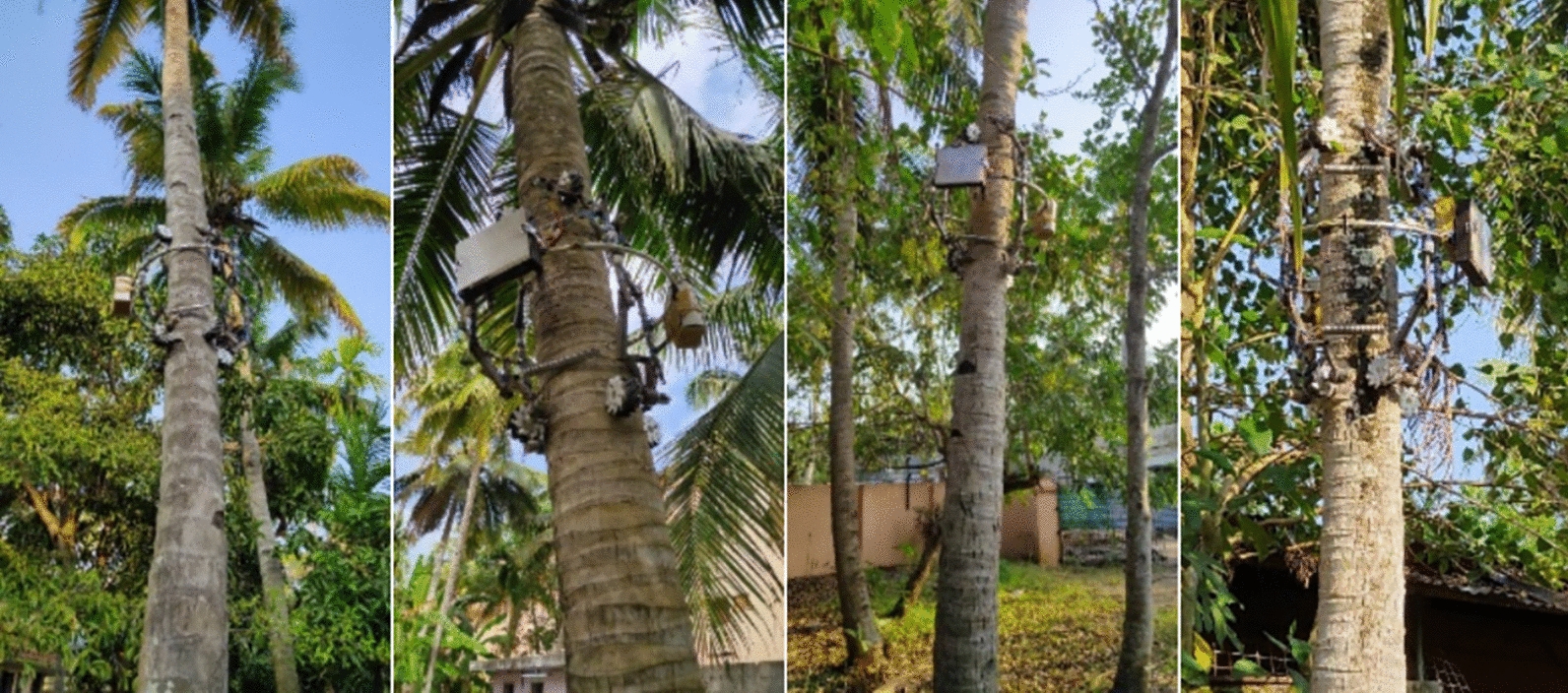
Measure the coconut tree parameters using robotic coconut tree climber

The spatial growth characteristics of a tree refer to how a tree grows and occupies physical space within its environment. These characteristics can vary depending on the species of tree and its specific environmental conditions. This includes the height of the tree, spread of the foliage, crown shape, branching pattern, growth rate etc. As part of the spatial analysis, the diameter variation and growth direction are considered. Spatial growth characteristics predicted by the proposed model, classical rod theory and biomechanical growth model is compared with the spatial growth characteristics obtained from the tree parameters measured using the robotic coconut tree climber. The climber is measuring the coconut tree parameters from the DBH. Ten coconut trees with age of 40 are considered for evaluation. As given in the Fig. 10 the height, diameter variation and direction of growth are measured from the sensor data collected by the sensor system. As part the evaluation, the variation of the diameter and growth direction are compared with respect to height of the coconut tree.

#### Primary growth

**Fig. 11:**
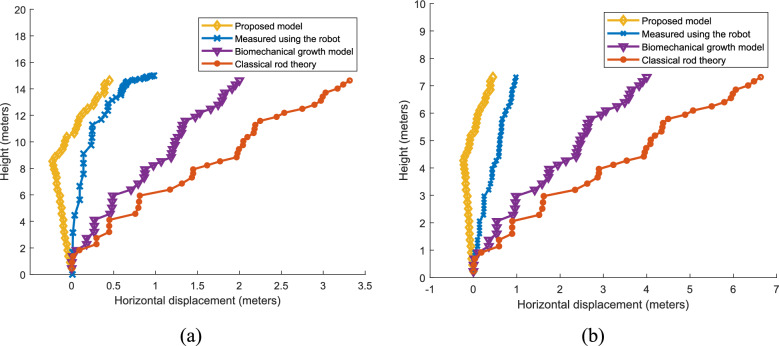
Comparison of the spatial growth (direction of growth) characteristics predicted by the proposed model for (**a**) WCT coconut palm (**b**) CGD coconut palm

In this section the spatial primary growth (direction of growth) predicted by the proposed model is compared with the real time data, classical rod theory and biomechanical growth model. In Fig. 11a. shows the growth direction with respect to height of the WCT coconut tree compared with the predicted characteristics by different models. The spatial growth characteristics predicted by the proposed method is closely aligned with the growth characteristic obtained from the tree parameters measured using the robotic coconut tree climber with a variance of 6.8. The variance is denoted in square meters. The growth model predicted by the biomechanical growth model and classical rod theory are showing the high deflection in X direction (parallel to ground) with respect to height with a variance of 40.3 and 127.3 respectively. Similarly, for the CGD coconut variety the characteristics are shown in Fig. 11b. Growth characteristics predicted by the proposed model and growth characteristics obtained from the real-time data are showing similar variation with respect to height (variance 10.3). The growth model predicted by the biomechanical growth model and classical rod theory shows the variance of 108.3 and 273.4 respectively with the real time data. It is crystal clear from the characteristics shown in Fig. 11a, b and the measured variance, the spatial primary growth characteristics predicted by the classical rod theory and biomechanical growth model are not suitable for coconut tree modeling.

#### Secondary growth

**Fig. 12:**
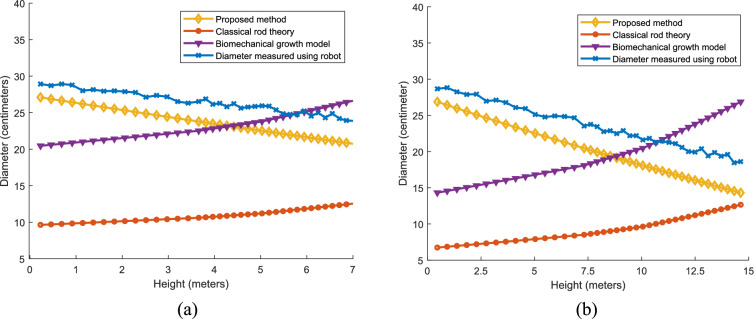
Comparison of the spatial growth (variation of diameter) characteristics predicted by the proposed model for (**a**) CGD coconut palm (**b**) WCT coconut palm

The spatial secondary growth characteristics predicted by the proposed model of WCT and CGD varieties of coconut tree are compared with the real time data, classical rod theory and biomechanics growth model. The diameter variation with respect to height of the CGD coconut tree variety is compared with the spatial secondary growth predicted by different models are given in Fig. 12a. The diameter variation predicted by the proposed method is closely knit with the growth characteristic obtained from the real time data with a variance of 82.4. The variance is denoted in square centimetres. The other two models are showing the increment in the diameter with respect to height. In normal case the diameter of the coconut tree reduces with respect to height. The growth predicted by the classical rod theory and biomechanical growth model show a variance of 198 and 678 respectively with the real time data. Similarly, spatial secondary growth characteristics of the WCT coconut variety is showed in Fig. 12b. Growth characteristics predicted by the proposed model (variance of 88.4) and real-time data show the decrement in the diameter with respect to height and the other two models showing the increment in the diameter with respect to height. The growth predicted by the classical rod theory and biomechanical growth model are showing a variance of 186.7 and 723.4 respectively with the real time data. The characteristics depicted in Fig. 12a, b as well as the observed variance make it obvious that the spatial primary growth characteristics predicted by the classical rod theory and biomechanical growth model are unsuitable for modeling coconut trees.

The temporal and spatial growth characteristics predicted by the proposed model demonstrates minimal variance from the real-time data, indicating high precision and accuracy. This stability underscores the reliability of proposed method, as it reflects minimal fluctuations from the actual values over time and height. In contrast, biomechanical growth model displays moderate variance, with noticeable fluctuations, implying a lower level of precision compared to the proposed method. Meanwhile, classical rod theory exhibits high variance and characterized by significant fluctuations, suggesting a lack of reliability in capturing the real time data. The experimental validation was conducted using a sample of 10 coconut trees, offering both advantages and limitations. The sample's representativeness depends on factors such as environmental diversity, geographical distribution, and growth variability, which influence the applicability of findings. If the trees were selected from varied conditions (e.g., different soil types, climates, and farming practices), the results may still hold relevance despite the small sample size. However, a limited dataset reduces statistical power, increasing the likelihood of potential bias and making it difficult to detect subtle trends. If all trees share common characteristics, the results may not generalize to broader coconut tree populations, and the presence of outliers could further impact the confidence in conclusions.

As in Table 2, the evaluation of the proposed model against the Classical Rod Theory and the Biomechanical Growth Model was conducted across different tree growth types, characteristics, and coconut tree types. For Primary Temporal Growth in WCT, the MAE, RMSE, and R^2^ values when compared to the Classical Rod Theory were 758.3, 758.3, and 0.0948, respectively, while for the Biomechanical Growth Model, they were 59.6, 59.6, and 0.6324. In the Primary Temporal Growth of CGD, the Classical Rod Theory comparison resulted in MAE 528.7, RMSE 528.7, and R^2^ 0.1019, whereas for the Biomechanical Growth Model, the values were 38.6, 38.6, and 0.6759. For Primary Spatial Growth in WCT, the Classical Rod Theory comparison yielded an MAE of 263.2, RMSE 263.2, and R^2^ 0.0732, while the Biomechanical Growth Model had 98.1, 98.1, and 0.1795 for the respective metrics. In the Primary Spatial Growth of CGD, the Classical Rod Theory comparison showed MAE 120.5, RMSE 120.5, and R^2^ 0.1040, while the Biomechanical Growth Model resulted in 33.5, 33.5, and 0.3090. For Secondary Temporal Growth in WCT, the Classical Rod Theory showed an MAE of 448.1, RMSE 448.1, and R^2^ 0.1040, while the Biomechanical Growth Model exhibited significantly higher values, with an MAE of 707.0, RMSE 707.0, and R^2^ 0.0679. These variations indicate that the proposed model aligns more closely with the Biomechanical Growth Model in some cases, while in others, it deviates significantly, emphasizing the need for further validation with larger datasets.

**Table 2:** Performance Evaluation of the Proposed Model Against Classical Rod Theory and Biomechanical Growth Model Across Different Growth Conditions

Tree growth	Characteristics	Coconut tree type	MAE (Classical vs Proposed)	RMSE (Classical vs Proposed)	RÂ^2^ (Classical vs Proposed)	MAE (Biomechanical vs Proposed)	RMSE (Biomechanical vs Proposed)	RÂ^2^ (Biomechanical vs Proposed)
Primary	Temporal	WCT	758.3	758.3	0.094756	59.6	59.6	0.632392
		CGD	528.7	528.7	0.101939	38.6	38.6	0.675873
	Spatial	WCT	263.2	263.2	0.073224	98.1	98.1	0.179495
		CGD	120.5	120.5	0.103981	33.5	33.5	0.308998
Secondary	Temporal	WCT	448.1	448.1	0.10403	707	707	0.067904
		CGD	260.5	260.5	0.126968	439.6	439.6	0.078333
	Spatial	WCT	115.7	115.7	0.658543	595.7	595.7	0.228038
		CGD	98.3	98.3	0.722784	635	635	0.229468

## Conclusion

This paper discussed about a growth model of coconut tree based on abiotic factors and modified Cosserat rod theory. Primary growth is modeled using gravitropism, the availability of sunlight and the effect of wind. The effect of sunlight is modeled by calculating the total available sunlight and the effect of wind is modeled by using cantiliver approximation method. The secondary growth is modeled using the diameter variation of the coconut tree trunk. In addition the diameter at breast height is included as an input parameter in the secondary growth model to accommodate the variations due to cultivation practices. The temporal growth characteristics and spatial growth characteristics for the WCT and CGD coconut varieties predicted by the proposed model is compared with the classical rod theory, and biomechanical growth model. A robotic coconut tree climber is developed to measure the growth characteristics of coconut trees. The sensor system placed in the robotic coconut tree climber precisely measured the coconut tree parameters. The temporal and spatial growth characteristics predicted by the proposed model are compared with the real time data, classical rod theory and biomechanical growth model. Compared to classical rod theory and biomechanical growth model, the proposed model shows least variance with the real time data.

. As mentions in the Table 3, the findings of the experiment suggest that the proposed model is more suitable for coconut trees compared to existing growth models, due to the unique morphology of the coconut tree. The two assumptions that are used in the model are this work's limitations. One is considering the DBH to accommodate the variations in the coconut tree due to the cultivation practises. DBH is insufficient to give all the variations in the tree growth due to cultivation practises. The second assumption is in the geometry of the coconut tree trunk. While modeling the secondary growth, to determine the geometrical characteristics, the coconut tree trunk is taken as the conical frustum. The coconut tree trunk may really be inclined, have anomalous diameter changes, etc. The proposed model demonstrates significant performance improvements over both the Classical Rod Theory and the Biomechanical Growth Model. On average, it reduces the Mean Absolute Error (MAE) by 85.00% compared to the Classical Rod Theory and by 82.43% compared to the Biomechanical Growth Model. The highest improvement is observed in the spatial growth characteristics of the CGD coconut tree type, where the MAE reduction reaches up to 96.27% compared to the Classical Rod Theory. Similarly, in comparison to the Biomechanical Growth Model, the highest MAE reduction is 96.55% in the spatial growth characteristics of the WCT coconut tree type. These results highlight the effectiveness of the proposed model in achieving more accurate predictions for both primary and secondary growth characteristics.

**Table 3:** Assessing the variance between growth characteristics predicted by various models and the real-time data

Tree growth	Characteristics	Coconut tree type	Classical rod theory	Biomechanical growth model	Proposed model
Primary	Temporal	WCT	797	98.3	38.7
		CGD	557.9	67.8	29.2
	Spatial	WCT	273.4	108.3	10.2
		CGD	127.3	40.3	6.8
Secondary	Temporal	WCT	473.4	732.3	25.3
		CGD	278.8	457.9	18.3
	Spatial	WCT	198	678	82.3
		CGD	186.7	723.4	88.4

Coconut tree modeling has diverse applications in precision agriculture, automated harvesting, tree health monitoring, climate change analysis, urban planning, and the biomass industry, helping optimize yield, resource management, and sustainability. It also plays a crucial role in genetic research, disaster preparedness, and risk assessment, enabling advancements in robotics, environmental conservation, and industrial applications for improved productivity and resilience. Expanding the dataset (number of trees consider for evaluation) in future research can strengthen the conclusions and validate the model’s effectiveness across diverse conditions. In future work, A sensitivity analysis may be incorporated to assess how variations in abiotic factors impact model predictions, enhancing the model's reliability. Future studies will explore additional factors influencing coconut growth, including climate conditions, soil type, soil pH, nutrient availability, water accessibility, variety differences, tree age, maturity stage, and fertilization practices. Integrating these variables will provide a more comprehensive and adaptive growth model, improving its applicability across diverse environments.

## Data Availability

The data that support the findings of this study are available from the corresponding author upon reasonable request.

## Appendix

**Table:**

Symbol	Implication
$k\left(t\right)$	Height with respect to time
$r$	Material parameter
${s}^{0}$	Base curve used to represent the position of the material in the Euclidean space
${f}_{1}^{0}$,${f}_{2}^{0}$	Orthonormal basis function for the orientation of the material in the Euclidean space
${a}_{v}$	Apical growth velocity
$y,w$	Strain functions
${y}^{0}$, ${w}^{0}$	initial strain vectors
${y}_{1}$, ${y}_{2}$	Bending strain
${y}_{3}$	Torsion strain/twist
${w}_{1}{, w}_{2}$	Shear strain
${w}_{3}$	Dilatation (value must be greater than zero)
${\gamma }^{0}\left(r\right)$	Torsion
${f}_{p}$	Preferential growth direction
$q(r,t)$	Contact couple vector
${\theta }_{S}$	Deflection in tree growth due to sunlight
$\theta$	Deflection in tree growth (without considering the effect of sunlight)
${\theta }_{p}^{f}$	The final deflection of tree trunk after considering the physical forces
$\beta$	The curvature of coconut tree
$Y$	Young’s modulus
$I$	Moment of inertial of the coconut tree trunk
$F$	Force acting due to wind
$D$	Beam deflection
${B}^{\prime}$	Bending moment
${{f}_{p}}^{*}$	Preferential growth direction due to sunlight and wind
${\theta }_{w}$	Deflection in tree growth due to wind
$x$	Radius of the Coconut tree
${V}^{r}$	Radial growth velocity
${A}_{r}$	Area of the cross section
${\rho }^{\prime}$	Mass density
${\rho }^{{\prime}{\prime}{\prime}}$	Areal mass density
${P}^{\prime}$	Pressure
${v}^{0}$	Original volume
${v}^{n}$	New volume
$d{v}^{\prime}$	Difference in volume
$B$	Bulk modulus
$g$	Acceleration due to gravity
